# Bovine Adenovirus-3 Tropism for Bovine Leukocyte Sub-Populations

**DOI:** 10.3390/v12121431

**Published:** 2020-12-12

**Authors:** Sugandhika Khosa, Maria Bravo Araya, Philip Griebel, Natasa Arsic, Suresh K. Tikoo

**Affiliations:** 1 VIDO-InterVac., 120 Veterinary Road, University of Saskatchewan, Saskatoon, SK S7N 5E3, Canada; sugandhika_khosa@yahoo.co.in (S.K.); Maria.bravo@pds.usask.ca (M.B.A.); Philip.Griebel@usask.ca (P.G.); Natasa.arsic@usask.ca (N.A.); 2 Vaccinology & Immunotherapeutics Program, School of Public Health, University of Saskatchewan, Saskatoon, SK S7N 5E3, Canada

**Keywords:** bovine adenovirus-3, tropism, leukocytes, chimeric pIX, RGD motif, EYFP

## Abstract

A number of characteristics including lack of virulence and the ability to grow to high titers, have made bovine adenovirus-3 (BAdV-3) a vector of choice for further development as a vaccine-delivery vehicle for cattle. Despite the importance of blood leukocytes, including dendritic cells (DC), in the induction of protective immune responses, little is known about the interaction between BAdV-3 and bovine blood leukocytes. Here, we demonstrate that compared to other leukocytes, bovine blood monocytes and neutrophils are significantly transduced by BAdV404a (BAdV-3, expressing enhanced yellow green fluorescent protein [EYFP]) at a MOI of 1–5 without a significant difference in the mean fluorescence of EYFP expression. Moreover, though expression of some BAdV-3-specific proteins was observed, no progeny virions were detected in the transduced monocytes or neutrophils. Interestingly, addition of the “RGD” motif at the C-terminus of BAdV-3 minor capsid protein pIX (BAV888) enhanced the ability of the virus to enter the monocytes without altering the tropism of BAdV-3. The increased uptake of BAV888 by monocytes was associated with a significant increase in viral genome copies and the abundance of EYFP and BAdV-3 19K transcripts compared to BAdV404a-transduced monocytes. Our results suggest that BAdV-3 efficiently transduces monocytes and neutrophils in the absence of viral replication. Moreover, RGD-modified capsid significantly increases vector uptake without affecting the initial interaction with monocytes.

## 1. Introduction

Adenoviruses are double-stranded DNA viruses, which offer several advantages as vaccine-delivery vectors in terms of efficacy, safety, and stability [1]. Recombinant adenoviral vectors have also been widely used for in vivo gene transfer because of their ability to enter many different cell types and efficiently express transgenes [2]. Although adenoviruses have been used to deliver vaccine antigens to cattle [1], we have focused on developing species-specific adenoviruses as vaccine-delivery vehicles [3]. Earlier, molecular characterization of bovine adenovirus 3 (BAdV-3) [4] led to the development of BAdV-3 as a vaccine vector for immunizing cattle [5,6,7,8]. Although two immunizations (prime and boost) with a BAdV-3-vectored vaccine induced protective immunity in calves [9], an ideal BAdV-3-vectored vaccine would induce protection following a single immunization [6] to avoid vaccine interference by acquired immune responses to the vector.

While intranasal immunization of calves with recombinant BAdV-3 expressing a truncated form of bovine herpesvirus 1 (BHV-1) glycoprotein gD induced protection against BHV-1 challenge, [9] parental immunization with the same recombinant BAdV-3 did not induce protective immune responses in calves against BHV-1 challenge [10]. The induction of protective immune responses following intranasal immunization of calves could be due, in part, to more efficient cell entry into respiratory mucosal epithelial cells.

One approach to increase the immunogenicity of the BAdV-3 vector-delivered vaccines may include the targeting of the BAdV-3 vector to antigen-presenting cells, such as dendritic cells (DCs). This could improve the efficiency of antigen presentation to naïve T cells. However, altering the tropism of BAdV-3 for a specific immune cell population requires knowledge of the normal tropism of BAdV-3 for bovine leukocyte subpopulations. Here, we determined the interaction of recombinant BAV304a with different subpopulations of bovine leukocytes and evaluated the transduction efficiency of a capsid-modified BAdV-3 with various bovine leukocyte subpopulations.

## 2. Materials and Methods

### 2.1. Cell Lines and Virus

Madin-Darby bovine kidney (MDBK) cells were grown in minimum essential medium (MEM) (Sigma-Aldrich Canada, Oakville, ON, Canada) supplemented with 10% fetal bovine serum (FBS) (Seracare, Life Sciences, Gaithersburg, MD, USA), 10 mM HEPES buffer (Life Technologies Inc., Burlington, ON, Canada), 0.1 mM non-essential amino acids (NEAA) (Life Technologies Inc., Burlington, ON, Canada), and 50 µg/mL gentamicin (GE Healthcare - HyClone Laboratories Inc., Logan, UH, USA). Recombinant BAV304a (BAdV-3 expressing enhanced yellow fluorescent protein inserted in the E3 deleted region under the control of CMV IE promoter) [11] and BAV888 (this study) were propagated in MDBK cells in MEM supplemented with 2% FBS. Virus was purified by cesium chloride density-gradient centrifugation [12] and virus titer determined by TCID_50_ assay [13].

### 2.2. Culture Media and Reagents

All bovine blood leukocyte cultures were maintained in AIM-V serum-free lymphocyte medium (GIBCO, Life Technologies Corporation, Grand Island, NY, USA) containing 10% (*v*/*v*) heat-inactivated fetal bovine serum (FBS; (GIBCO, Life Technologies Corporation, Grand Island, NY, USA). Unless otherwise indicated, the cultures were incubated at 37 °C in a humidified atmosphere with 5% CO_2_.

### 2.3. Monoclonal Antibodies

Primary monoclonal antibodies (MAbs) used to detect bovine leukocyte antigens (Table 1), isotype controls, and fluorochrome-conjugated secondary antibodies (Table 2) are listed.

### 2.4. BAdV-3 Protein-Specific Antibodies

Production and characterization of anti-BAdV-3 E1B small 19K protein [14], DNA-binding protein [15], 52K protein [16], and 100K protein [17] specific sera has been previously described. Anti-hexon sera recognizes a protein of 110 kDa in BAdV-3 infected cells [18]. Production and characterization of anti-BAdV-3 pIX sera has been described [19].

### 2.5. Plasmid Construction

To isolate recombinant BAdV-3, full-length BAdV-3 plasmid containing chimeric pIX was constructed following standard DNA manipulation techniques, described earlier [19].

#### 2.5.1. Plasmid pBAVNdA-10GS-CATRGD

Plasmid pBAVNdA plasmid [19] (containing E1A and E1B regions and protein pIX DNA sequence of the BAdV-3 genome followed by a 10GS linker sequence) was digested with *HpaI* -*XhoI* restriction enzymes. Oligonucleotides containing a cathepsin cleavage site [20] and Arg-Gly-Asp (RGD) sequence were annealed creating overhangs for *HpaI* and *XhoI* sites and ligated to the *HpaI-XhoI* digested plasmid pBAVNdA [19] DNA creating plasmid pBAVNdA-10GS-CATRGD.

#### 2.5.2. Plasmid pUC304a-CATRGD

Homologous recombination in *E. coli* BJ5183 [21] was performed between the *Sex*AI-*Pme*I digested plasmid pUC304a DNA (containing the genome of BAdV-3 with an enhanced yellow green fluorescent protein (EYFP) cassette in the E3 deleted region) [11], and an *Ase*1-*Not*1 11 kb DNA fragment of plasmid pBAVNdA-10GS-CATRGD, creating plasmid pUC304a-CATRGD.

### 2.6. Isolation of Recombinant BAdV-3

VIDO DT1 [11] cells in a 6-well plate (1 × 10^6^ cells/well) were transfected with 4–6 µg/well of plasmid pUC304a-CATRGD DNA using Lipofectamine 2000 (Invitrogen Canada Inc., Burlington, ON, Canada). At 6 h post-transfection, the medium was replaced with fresh MEM containing 2% FBS (MEM; Sigma-Aldrich, St. Louis, MO, USA); (FBS; Sigma-Aldrich, St. Louis, MO, USA) and the cells were observed daily under the microscope for visible cytopathic effects (CPE). After 15 to 20 days post transfection, the cultures with visible CPE were collected, freeze-thawed three times to lyse the cells, and used for purification of recombinant virus designated as BAV888 [12,13].

### 2.7. Isolation of Bovine Blood Leukocytes

All animal experimental procedures were approved by the University of Saskatchewan University Animal Care Committee (AUP Number 20170015; 1 April 2017) following guidelines approved and permitted by the Canadian Council on Animal Care.

#### 2.7.1. Isolation of Bovine Peripheral Blood Mononuclear Cells (PBMCs)

Blood was collected by venipuncture from castrated male Holstein calves between 9 and 12 months of age, housed at the VIDO-InterVac facility (Saskatoon, SK, Canada), using 7.5% ethylenediaminetetra–acetic acid (EDTA, Sigma-Aldrich) as an anticoagulant. The peripheral blood mononuclear cells (PBMCs) were purified using a Percoll density gradient as described previously [22].

#### 2.7.2. Bovine Polymorphonuclear (PMN) Cell Isolation

PMNs were purified using the protocol as described [23]. Briefly, whole blood collected in 7.5% EDTA was centrifuged at 1400× *g* for 20 min, the buffy coat was removed, and the red blood cell (RBC) fraction was re-suspended in an equal volume of phosphate buffered saline (PBS; pH 7.2). The cell suspension was centrifuged at 1400× *g* for 20 min. Medium and the cells were aspirated leaving the bottom half of the RBC fraction, which contains the PMN leukocyte population. The RBCs were lysed by adding 5–10 mL of the RBC/PMN fraction to 40 mL of a lysis solution (distilled water with 0.17 M NH4Cl, 1 mM KHCO3, and 0.11 mM EDTA; pH 7.3) followed by three washes with PBSA.

#### 2.7.3. Bovine Monocyte Isolation

The monocytes (CD14+) were isolated from PBMCs as described previously [22]. Alternately, high-speed cell sorting of monocyte (CD14^+^CD11c^+^ cells) was performed by first labelling 3 × 10^8^ bovine PBMC (1 × 10^8^ cells/mL) in PBS, with 5 µL/mL each of anti-bovine CD11c and anti-bovine CD14 monoclonal antibodies (Table 1). The cells were incubated for 20 min at 4 °C, with gentle mixing every 10 min, before pelleting the cells and washing twice with ice-cold PBS. The cell pellet was re-suspended in 3 mL PBS and incubated in the dark at 4 °C with 5 µL/mL each of goat anti-mouse IgG1-PE and goat anti-mouse IgM-APC. After a 20-min incubation, the cells were washed twice with ice-cold PBS and re-suspended in ice-cold PBS at a final concentration of 1 × 10^8^ cells/mL. The cells were filtered through a 35-um cell strainer (BD Falcon Franklin Lakes, NJ, USA) into 12 × 75 mm polystyrene round bottom tubes (BD Falcon Franklin Lakes, NJ, USA) and viable CD14^+^CD11C+ monocytes were sorted with a MoFlo XDP (Beckman Coulter Inc., Loveland, CO, USA) high-speed cell sorter. PE and APC fluorescence signals were gathered through 575/25 and 670/30 band-pass filters, respectively. The first sort region (R1) was defined by dot scatter plots exhibiting forward light scatter (FSC) and side scatter (SSC) and region parameters were set to exclude dead cells and the cell debris. The second sort region (R2) was defined by dot plots exhibiting FSC-Height and FSC-Width. The parameters were set to exclude doublets or larger cell clumps. A gated region was established on the basis of CD14 and CD11c co-expression. The final sort conditions were 60 psi sheath pressure, 70 µm nozzle, differential pressure of 0.3 psi, and a sort rate of 18,000–22,000 events/s. Sterile 12 × 75 mm polypropylene round bottom tubes (VWR International, Mississauga, ON, Canada) kept on ice were used to collect sorted monocytes. The sorted monocytes were pelleted and suspended in ice-cold AIM-V medium (Invitrogen, Burlington, ON, Canada) supplemented with 10% fetal bovine serum (GIBCO, Life Technologies Corporation, Grand Island, NY, USA) after every 30 min sort interval.

#### 2.7.4. Bovine Dendritic Cell Isolation

For dendritic cell sorting, 1 × 10^8^ PBMCs were re-suspended in 1 mL PBS and incubated for 20 min at 4 °C with 4 µL/mL anti-bovine CD14 and 4 µL/mL anti-bovine CD209 Mab [24,25]. The cells were washed twice with ice-cold PBS before re-suspending in 3 mL PBS and incubating with 5 µL/mL goat anti-mouse IgG1-APC and 3 µL/mL goat anti- mouse IgG2a-PE. After a 20 min incubation, the cells were washed twice with ice-cold PBS before re-suspending in ice-cold PBS at a final concentration of 1 × 10^8^ cells/mL. The PBMCs were then filtered through 35 µm cell strainer capped 12 × 75 mm polystyrene round bottom tubes (BD Falcon Franklin Lakes, NJ, USA). Finally, PBMCs were subjected to high-speed cell sorting with a MoFlo XDP (Beckman Coulter Inc., Loveland, CO, USA) and PE and APC fluorescence were collected through 575/25 and 670/30 band-pass filters, respectively. The first sort region (R1) was defined by dot scatter plots exhibiting forward light scatter (FSC) and side scatter (SSC) to exclude dead cells and debris. The second sort region (R2) was defined by dot plots exhibiting FSC-Height and FSC-Width to exclude doublets or large cell clumps.

The DCs were sorted with a region that included CD209^+^ but excluded CD14^+^ cells as described previously [26]. R1 and R2 regions were also used when sorting monocytes based on co-expression of CD14 and CD209. The conditions for the sort were: 60 psi sheath pressure, 70 µm nozzle, differential pressure of 0.3 psi, and a sort rate of 18,000–22,000 events/s. Sterile 12 × 75 mm polypropylene round bottom tubes (VWR international, Mississauga, ON, Canada) kept on ice were used to collect the sorted c dendritic cells and after every 30 min sort interval sorted cells were pelleted and suspended in ice-cold AIM-V medium (Invitrogen Canada Inc., Burlington, ON, Canada) supplemented with 10% fetal bovine serum (GIBCO, Life Technologies Corporation, Grand Island, NY, USA).

### 2.8. Transduction of Bovine Leukocytes by BAV304a

Bovine PBMCs (0.5 × 10^6^ cells/well) or PMNs cells (0.5 × 10^6^/well) were transduced by adding BAV304a at a multiplicity of infection (MOI) of 1, 2, 5, or 10. After incubating the cells for 18 h at 37 °C, PBMCs and PMN cells were harvested using 0.5% trypsin, re-suspended in PBS (pH 7.4) fixed with 2% paraformaldehyde, and analyzed with a FacsCalibur using Cell Quest acquisition and analysis software. Each sample was analyzed by capturing a minimum of 10,000 events.

### 2.9. Transduction of Bovine PBMC Subpopulations by BAV304a

To determine which bovine PBMC subpopulations were transduced by BAV304a, about 2 × 10^6^ bovine PBMC were incubated for 18 h with BAV304a at an MOI of 2. Then, PBMCs were labeled with anti-bovine CD3 (pan-T cell marker), anti-bovine CD14, and anti-bovine CD11c (monocyte markers), anti-bovine CD21 (B cell marker), and anti-bovine CD335 (innate lymphoid cell [ILC] marker) MAbs. Labeling with MAbs was detected with by adding fluorochrome-conjugated secondary antibodies (goat anti–mouse IgG1-PE or goat anti-mouse IgM-APC) (Table 1). Finally, PBMCs were fixed with 2% paraformaldehyde and stored in the dark at 4 °C until analyzed with a FacsCalibur using Cell Quest acquisition and analysis software. Each sample was analyzed by capturing a minimum of 10,000 events.

### 2.10. Western Blot Analysis

Western blot analysis was performed as described [13]. Briefly, sorted bovine monocytes or PMN cells were seeded in 6-well plates, and either mock-infected or infected with BAV304a at a MOI of 2. After 48 h of incubation, the cells were collected and analyzed by Western blot using BAdV-3 protein-specific antibodies.

### 2.11. Virus Titer Assay

The titer of BAV304a recovered from transduced bovine monocytes or PMNs cells was determined using the 50% tissue culture infectious dose (TCID_50_) assay [12,13]. Purified monocytes or PMNs cells were incubated with BAV304a at a (MOI of 2. At 48 h post-infection, the cells were harvested, subjected to five freeze-thaw cycles and the virus in cell lysates was titrated using MDBK cells. Virus titer was determined by counting the number of fluorescent foci/well using a fluorescence microscope and expressed as TCID_50/mL_ [12,13].

### 2.12. qRT-PCR

About 5 × 10^6^ purified bovine monocytes (CD14^+^) were transduced with BAV304a or BAV888 at MOI of 1. At 16 h post-infection, the supernatant from each well was collected and centrifuged at 300× *g* for 8 min to pellet any cells in suspension. The RNA was extracted using the RNeasy mini kit (Qiagen, Hilden, Germany) as per manufacturer’s instructions. The RNA samples were analyzed for quality and quantity using the Agilent 2100 Bioanalyzer (G2938B, Agilent Technologies, Santa Clara, CA, USA). Samples with a RNA Integrity Number (RIN) above 7 were used to synthesize cDNA using the QuantiTect Reverse Transcription Kit (Qiagen, Hilden, Germany) as per manufacturer’s instructions.

The samples were analyzed with the iCycler iQ PCR detection system (Bio-Rad Laboratories (Canada) Ltd., Mississauga, ON, Canada). Each reaction contained 10 ng of cDNA, 0.5 µL of each primer pair (Table 3) at a concentration of 10 µM (Table 3), 0.5 µL of nuclease-free water (Sigma-Aldrich, St. Louis, MO, USA) and 9 µl of PerfeCTa SYBR Green SuperMix for iQ (Quanta BioSciences Inc., MD, USA). The reaction was 1 cycle at 50 °C for 2 min; 1 cycle at 95 °C for 30 s; 45 cycles at 95 °C for 15 s, 60 °C for 30 s and 72 °C for 30 s. β-actin was used as the reference gene, and all reactions were performed in duplicate.

### 2.13. qPCR Assay for Viral Quantification

A total of 2 × 10^6^ purified bovine monocytes (CD14+) were transduced with BAV304a or BAV888 at MOI of 1. At 16 h post-infection, the cells were collected by centrifugation at 300× *g* for 7 min and resuspended in PBS. The DNA was extracted from the cells using the DNA Blood and Tissue kit (Qiagen, Hilden, Germany) as per the manufacturer’s instructions. Finally, the extracted DNA was subjected to quantitative PCR using the iCycler iQ PCR detection system (Bio-Rad Laboratories (Canada) Ltd., Mississauga, ON, Canada), using BAdV-3 pVIII-specific primers (Table 3). Viral copy numbers were calculated using a standard curve for wild-type BAdV-3 as described earlier [27].

### 2.14. Data Analysis

Data presented were parametric and were analyzed with GraphPad Prism 7 software (GraphPad Software, San Diego, CA, USA). Differences between viruses were assessed with Student *t*-tests and one-way ANOVA was used to determine differences among different MOI in flow cytometry experiments. Differences were considered significant with a *p*-value of less than 0.05.

## 3. Results

### 3.1. Transduction of Bovine PBMCs by BAV304a

**Figure 1:**
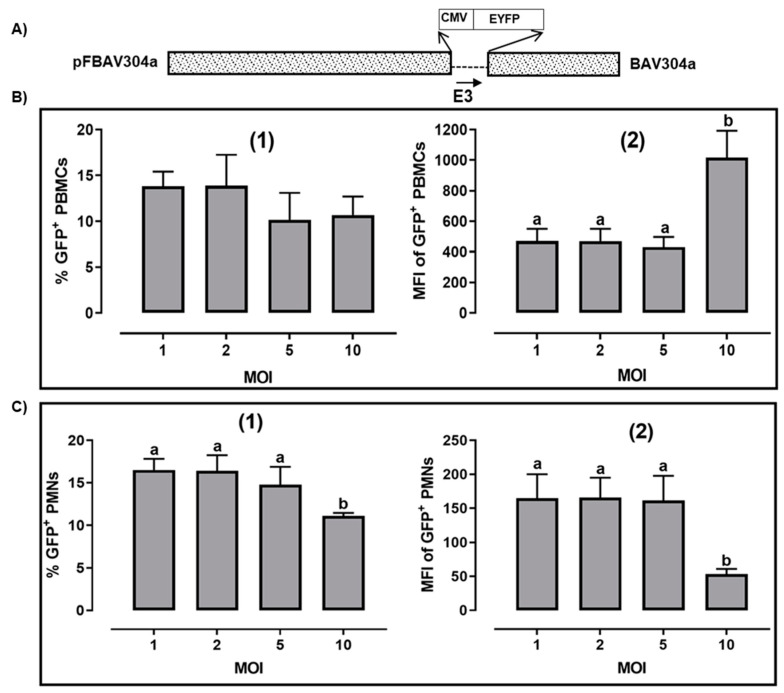
Transduction of bovine cells by BAV304a. (**A**) Schematic diagram of BAV304a genome. Human cytomegalovirus immediate early gene promoter (CMV) and enhanced yellow fluorescent protein gene (EYFP) were inserted in Early (E)-3 region. The direction of transcription is shown by an arrow. Dashed line represents deleted region. The name of the plasmid is on the left and name of the virus is indicated on the right. Purified bovine peripheral blood mononuclear cells (PBMCs) (**B**) or purified bovine PMNs (**C**) were infected with increasing MOI of CsCl purified BAV304a. At 18 h post infection, the cells were collected and analyzed to quantify the percentage of cells expressing EYFP (subpanel 1) and the mean florescent intensity (MFI) of EYFP expression in transduced cells (subpanel 2).

To determine the optimum BAV304a (Figure 1A) [11] MOI required to transduce bovine leukocytes, PBMCs isolated from four animals were either mock-infected or infected with BAV304a at a MOI of 1, 2, 5, or 10. After incubation for 18 h at 37 °C, the infected cells were trypsinized, fixed with 2% paraformaldehyde, and fluorescence (FL1) was analyzed by flow cytometry. The percentage of virus-transduced PBMCs was determined based on a detectable increase in mean fluorescence intensity (MFI) when comparing infected versus mock-infected cells. Between 10 and 15% of PBMCs had a detectable level of fluorescence exceeding the background fluorescence in mock-infected cells, irrespective of the MOI used (Figure 1B1. Analysis of mean fluorescence intensity (MFI) in the EYFP^+^ population revealed a relatively high level of EYFP expression in transduced cells at all MOIs (Figure 1B2, with MFI significantly (*p* < 0.05) increased at MOI of 10. These data are consistent with the suggestion that a limited population of PBMC are being transduced but within that population, the cells have the capacity to increase uptake of virus particles at the highest MOI. 

### 3.2. Transduction of Bovine PMN Cells by BAV304a

Purified PMN cells were consistently >98% viable as determined by trypan blue dye exclusion and contained less than 0.1% mononuclear cells. Bovine PMNs, which contain both neutrophils and eosinophils, were isolated from four different animals and were either mock-infected or infected with BAV304a at a MOI of 1, 2, 5, or 10. After incubation for 18 h at 37 °C, the infected cells were trypsinized, fixed in 2% paraformaldehyde and fluorescence (FL1) was analyzed by flow cytometry. As seen 10 to 18% of PMNs were transduced by BAV304a (Figure 1C1. There was no significant difference in either percent-transduced cells (Figure 1C1 or MFI (Figure 1C 2 when using an MOI of 1 to 5. However, there was a significant (*p* < 0.01) decrease in both percent-transduced PMNs (Figure 1C1 and MFI of EYFP expression in PMNs when using an MOI of 10 (Figure 1C2.

### 3.3. Transduction of PBMC Subpopulations by BAV304a

As less than 20% of PBMCs had detectable levels of EYFP expression (Figure 1B1), it was hypothesized that there may be selective uptake of BAV304a by specific leukocyte sub-populations. To address this question, monoclonal antibodies (Table 1) recognizing leukocyte differentiation antigens were used to identify individual PBMC lineages following incubation of PBMCs with BAV304a at an MOI of 2 for 18 h.

**Figure 2:**
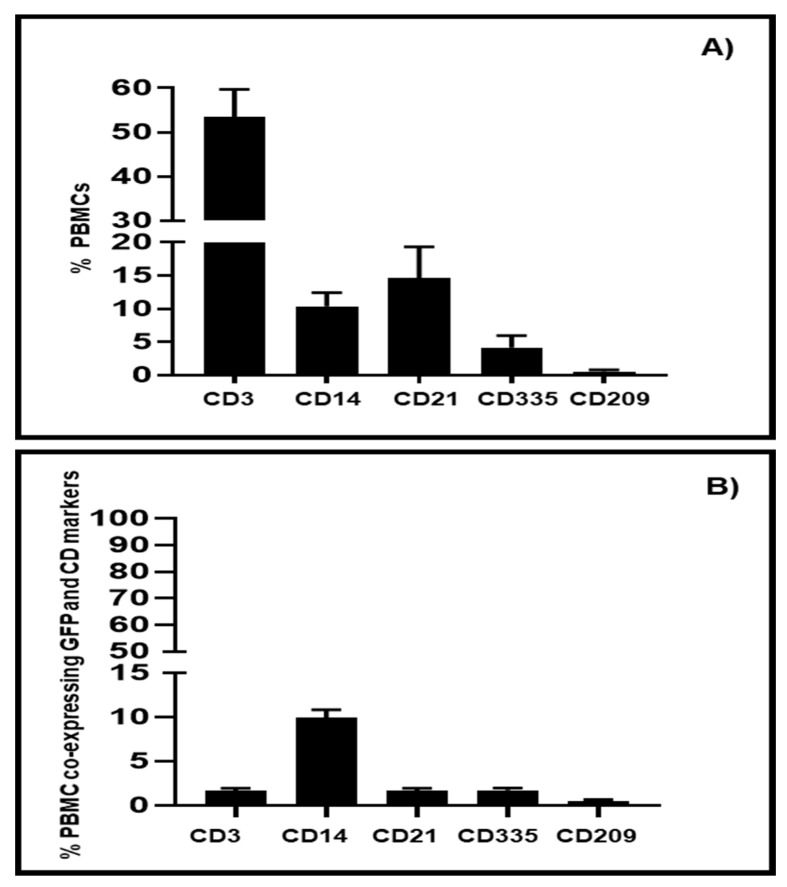
Transduction of leukocyte subpopulations by BAV304a. (**A**) Bovine PBMCs were isolated and labeled to quantify the percentage of cells within major leukocyte lineages: T cells (CD3^+^); monocytes (CD14^+^), B cells (CD21^+^); innate lymphoid cells (CD335^+^), and dendritic cells (CD209^+^). Data presented are the mean + 1 SD of values for PBMCs isolated from five animals. (**B**) Bovine PBMCs were transduced with BAV304a (MOI 2) for 18 h before labelling with leukocyte lineage-specific monoclonal antibodies and analyzed for co-expression of EYFP. Data presented are the mean + 1 SD of the percentage of cells within each leukocyte lineage expressing EYFP. Transduction experiment was repeated with PBMCs isolated from five animals.

Flow cytometric analysis indicated that isolated bovine PBMCs consisted of 52–59% CD3^+^ T cells, 9–12% CD14+ monocytes, 13–18% CD21^+^ B cells, 3–5% CD335^+^ ILCs, and 0.8% CD209^+^ dendritic cells (Figure 2A). Phenotypic analyses of EYFP^+^-transduced PBMCs revealed CD14^+^ monocytes were the primary population transduced by BAV304a with more than 90% CD14^+^ cells being GFP^+^ (Figure 2B). In contrast, less than 5% of other leukocyte lineages, including T cells (CD3^+^), B cells (CD21^+^), and ILCs- (CD335^+^), expressed detectable levels of GFP (Figure 2B). Interestingly, 1.5% of DCs (CD209^+^) were transduced with BAV304a (Figure 2B).

### 3.4. Viral Replication in Transduced Bovine Monocytes and PMNs Cells

**Figure 3:**
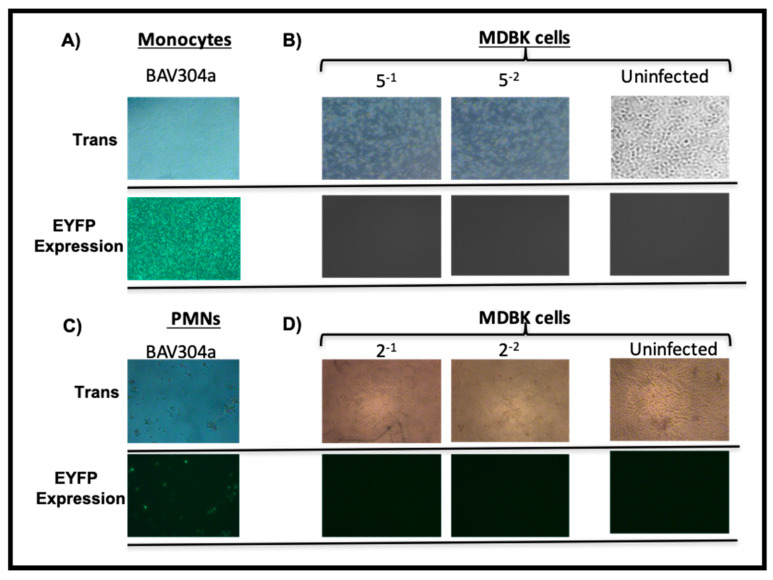
Replication of BAV304a in monocytes and PMNs. Purified CD14^+^ bovine monocytes (**A**) or bovine PMN cells (**C**) were infected with BAV304a at an MOI of 2. At 48 h post-infection, the cells were examined by light microscopy (Trans) to confirm viability and with fluorescent microscopy (EYFP expression) to confirm transduction. The infected cells were collected, freeze-thawed and the virus in infected monocyte lysates (**B**) or infected PMNs lysates (**D**) was titrated using MDBK cells. Viral infection of MDBK cells was analyzed for cytopathic effect (CPE) with light microscopy (Trans) and expression of EYFP by fluorescent microscopy (EYFP expression). Results presented are representative of two independent experiments with three replicates cultures in each experiment. Five-fold serial dilutions of monocyte lysates and two-fold serial dilutions of PMN lysates were used for virus titration on MDBK cells. Magnification in all images is 100×.

Bovine monocytes or PMN cells were efficiently transduced by BAV-304a as determined by EYFP expression. A high level of transduction could be due to either efficient BAV304a uptake by these cell populations or viral replication within cells. To determine if leukocytes were permissive for virus replication, purified monocytes (Figure 3A) or PMNs (Figure 3C) were incubated with BAV304a. After 48 h of incubation, the transduced monocytes (Figure 3B) or PMNs (Figure 3C) were harvested, freeze-thawed, and the lysate analyzed for progeny virions using MDBK cells in a TCID_50_ assay [13]. No expression of EYFP or visible cytopathic effect (CPE) was observed in MDBK cells following 14-day incubation with the cell lysate from BAV304a-infected monocytes (Figure 3B) or PMN cells (Figure 3D). Some cell death appeared because of the use of crude infected cell lysates. This suggested that BAV304a could not replicate in either bovine monocytes or PMN cells.

### 3.5. BAdV-3 Viral Gene Expression in Transduced Bovine Monocytes and PMN Cells

**Figure 4:**
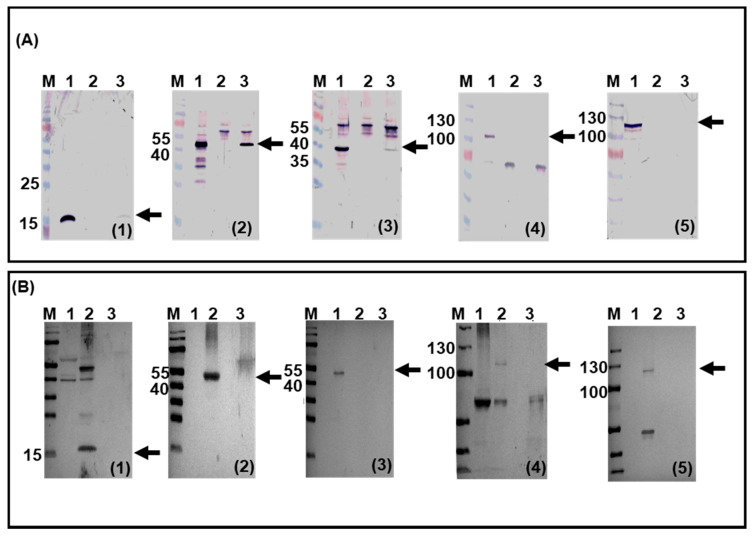
Viral protein expression in BAV304a-infected cells. Monocytes (**A**) and PMNs (**B**). The infected cell lysates were separated with 12% SDS-PAGE, transferred to nitrocellulose membranes and probed in Western blot using BAdV-3 anti-19K serum (subpanel **1**), BAdV-3 anti DBP serum (subpanel **2**), BAdV-3 anti-52Kserum (subpanel **3**), BAdV-3 anti-hexon serum (subpanel **4**), and BAdV-3 anti-100K serum (subpanel **5**). BAV304a-infected MDBK cells (panel **A**, lanes 1; panel **B** lanes 2); BAV304a-infected monocytes (panel **A**, lanes 3) or PMNs (panel **B**, lanes 2); mock-infected monocytes (panel **A**, lanes 2) or PMNs (panel **B**, lanes 1); molecular weight markers (M) in kDa are shown on the left.

To determine whether an abortive viral infection occurred in bovine leukocytes, the expression of specific BAdV-3 proteins was analyzed in transduced cells. Proteins in the lysates of infected monocytes were run on 12% SDS-PAGE, transferred to nitrocellulose and probed in Western blots using BAdV-3 protein-specific antisera. As seen in Figure 4A, expression of E1-encoded 19K protein (Figure 4A1- lane 1), E2-encoded DNA binding protein (DBP) (Figure 4A2- lane 1), and L1-encoded 52K protein (Figure 4A3- lane 1) were detected in BAV304a-infected MDBK cells. Interestingly, E1-encoded 19K Figure 4A1- lane 3), E2-encoded DNA binding protein (DBP) (Figure 4 A2- lane 3), and L1-encoded 52K protein (Figure 4A3- lane 3) were also detected in BAV304a-infected bovine monocytes. As expected, the late proteins, including hexon (Figure 4A4- lane 1) and 100K (Figure 4A5- lane 1), were detected in BAV304a-infected MDBKs. In contrast, neither hexon protein (Figure 4A4- lane 3) nor the 100K protein (Figure 4A5- lane 3) were detected in BAV304a-infected monocytes. None of the viral proteins could be detected when lysate from mock-infected MDBK cells were probed with the specific antisera (Figure 4A1–5- lane 2).

Western blot analysis of BAdV-3 proteins was also performed with the lysates from BAV304a infected bovine PMN cells. Expression of E1-encoded 19K protein (Figure 4B1- lane 2), E2-encoded DBP (Figure 4B2- lane 2), L1-encoded 52K (Figure 4B3- lane 2), L5-encoded hexon protein (Figure 4B4- lane 2), and L6-encoded 100K protein (Figure 4B5- lane 2) was detected in BAdV-3 infected MDBK cells. In contrast, no expression of E1-encoded 19K (Figure 4B1- lane 3), E2-encoded DBP (Figure 4B2- lane 3), or L1-encoded 52K (Figure 4B3- lane 3), L5-encoded hexon protein (Figure 4B4- lane 3), and L6-encoded 100K protein (Figure 4B5- lane 3) could be detected in BAV304a-transduced PMN cells or mock-infected MDBK cells (Figure 4B1 to 5- lane 1).

### 3.6. Isolation of Recombinant BAV888

**Figure 5:**
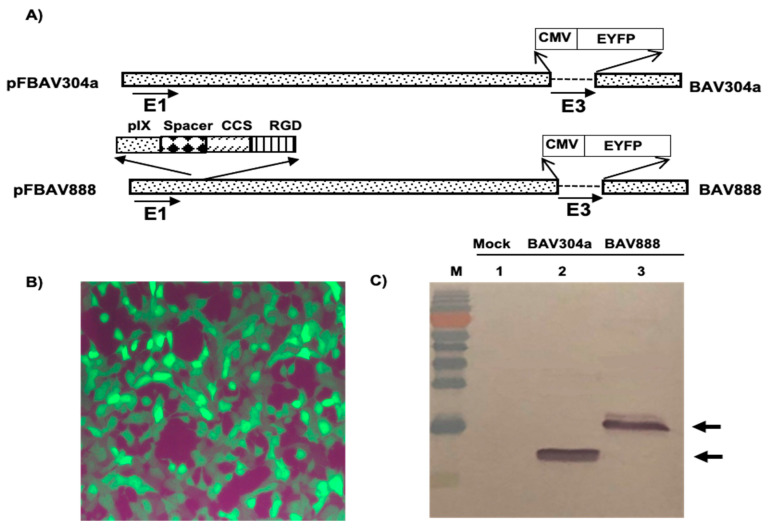
Construction and characterization of BAV888. (**A**) Schematic diagram of BAV304a and BAV888 genomes; cathepsin cleavage site (CCS); 10 repeats of glycine-serine (spacer), RGD amino acids (RGD). The shaded box represents the BAdV-3 genome sequence, while the dashed line in early (E)-3 region represents deletion in this region. Human cytomegalovirus immediate early gene promoter (CMV); enhanced yellow fluorescent protein (EYFP) gene. The direction of transcription is indicated with arrows. The name of each plasmid is indicated to the left and virus name is indicated to the right. (**B**) EYFP fluorescence. The cells were infected with BAV888 and analyzed by Leica TC5 SP5 immunofluorescence microscope (Magnification 400×)**.** (**C**) Expression of pIX in BAV304a and BAV888 infected cells. The proteins from the lysates of mock-infected cells (lane 1), BAV304a infected cells (lane 2) or BAV888 infected cells (lane 3) were separated with 15% SDS-PAGE, transferred to a nitrocellulose membrane and probed in Western blot using anti-pIX antiserum. The pIX protein is indicated by arrows. Molecular weight markers (M).

The outer cytoplasmic membrane of leukocytes, including antigen-presenting cells, are rich in integrins which play important roles in intercellular interactions and interactions with tissue structures [28,29]. The addition of an “RGD” motif at the C-terminus of BAdV-3 minor capsid protein pIX could enhance the ability of the virus to attach to and enter integrin positive cells [19,30]. We investigated whether the addition of an “RGD” motif would alter the leukocyte tropism of BAV304a (E3 deleted BAdV-3 containing CMV-EYFP cassette inserted in E3 region; Figure 5A) [11]. A plasmid, pUC304a-CATRGD (Figure 5A), was constructed containing a full-length BAV304a genomic clone expressing chimeric pIX (C-terminus of pIX fused in frame to a cathepsin cleavage site (ccs) and a RGD motif). Transfection of VIDO DT1 cells (cotton rat lung [CRL] expressing I-SceI protein) [11] with plasmid pUC304a-CATRGD DNA produced cytopathic effect (CPE) in 15–20 days. The cells showing CPEs and EYFP expression (Figure 5B) were harvested, freeze-thawed three times, and the recombinant virus named BAV888 was propagated in MDBK cells before purifying by CsCl banding.

The identity of the recombinant BAV888, was first determined by sequencing viral DNA. The expression of recombinant protein IX was analyzed by Western blot using anti-pIX antiserum [19]. The anti-pIX antibody detected a 14 kDa protein in BAV304a infected cells (Figure 5C; Lane 2) and a 16 kDa protein in BAV888-infected cells (Figure 5C; Lane 3). No protein reacted with the anti-pIX antibody when probing the lysate from mock-infected cells (Figure 5C; Lane 1).

### 3.7. Transduction of PBMC Subpopulations by BAV888

**Figure 6:**
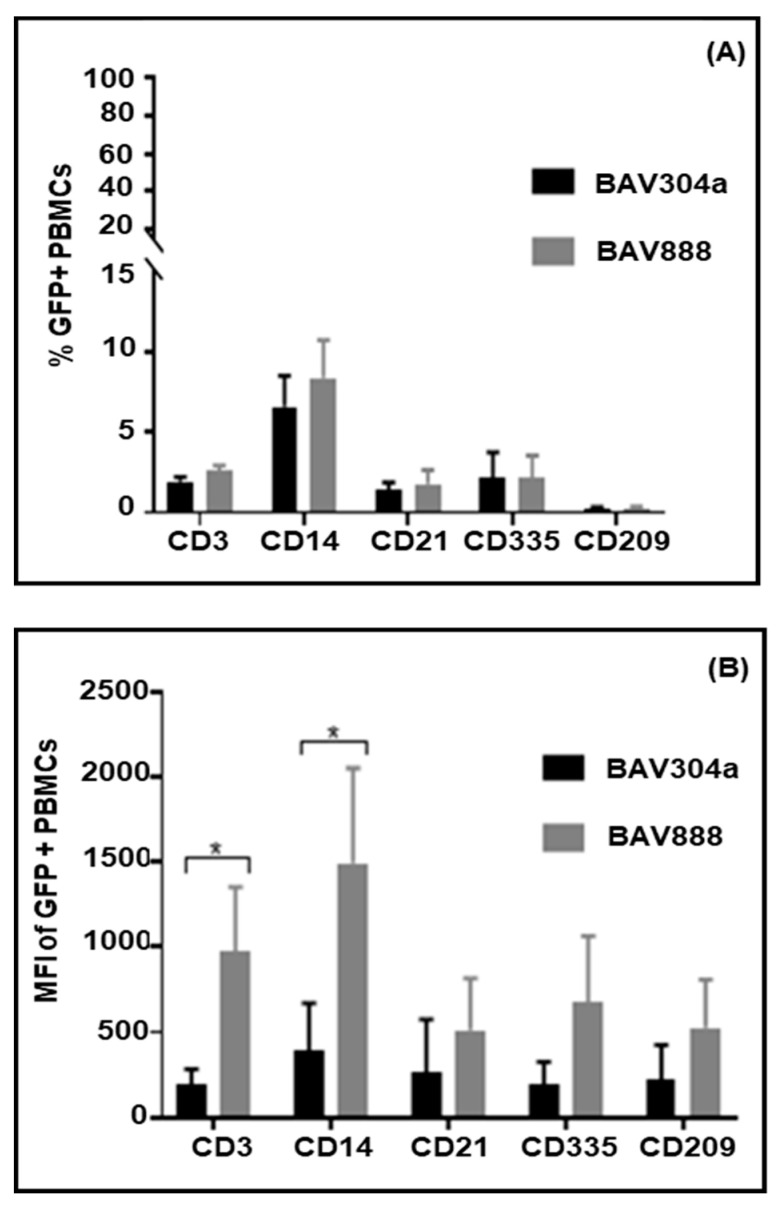
Comparison of bovine leukocyte transduction by BAV304a or BAV888. Bovine PBMCs were transduced for 16 h with either BAV304a or BAV888 (MOI = 1). Cells were labelled with lineage-specific monoclonal antibodies and analyzed for co-expression of GFP: T cells (CD3^+^), monocytes (CD14^+^), B cells (CD121^+^), innate lymphoid cells (CD335^+^), and DCs (CD209^+^). The percent GFP^+^ cells (panel **A**) and EYFP mean fluorescence intensity (MFI) (panel **B**) were analyzed for each PBMC lineage. Data presented are the mean + 1SD of values from PBMCs isolated from five animals. * Significant (*p* < 0.05) differences when comparing BAV304a or BAV888 transduction within a leukocyte lineage.

To determine if the RGD motif altered BAdV3 tropism isolated bovine PBMCs were transduced with either BAV304a or BAV888. PBMCs were incubated for 16 h with virus and before labelling with bovine leukocyte lineage-specific monoclonal antibodies. The labeled cells were analyzed by flow cytometry for co-expression of EYFP. When comparing PBMCs transduced by BAV304a or BAV388 there was no significant difference in the % of transduced monocytes (CD14^+^), ILCs (CD335^+^), DCs (CD209^+^), T cells (CD3^+^), or B cells (CD21^+^) (Figure 6A). In contrast, the mean fluorescence intensity (MFI) of EYFP expression was significantly (*p* < 0.05) greater in BAV888 versus BAV304a-transduced T cells (CD3^+^) and monocytes (CD14^+^) (Figure 6B).

### 3.8. Viral Genome and Transgene Expression in Transduced Bovine Monocytes (CD14^+^)

**Figure 7:**
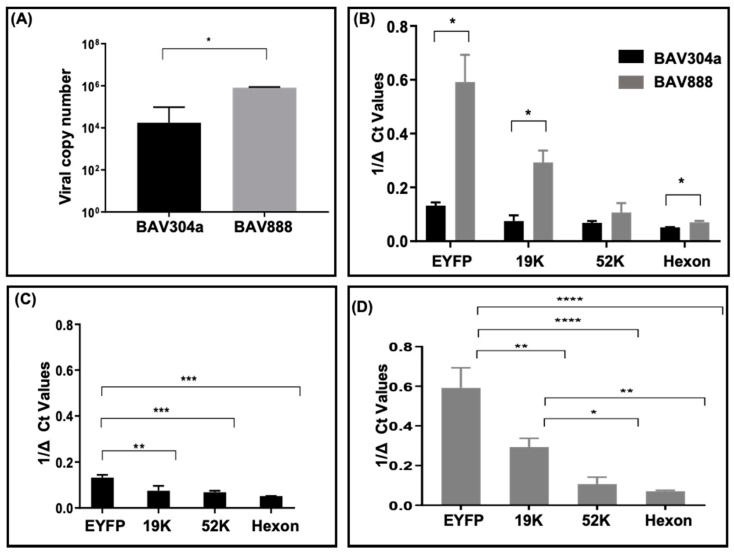
BAdV-3 genome copy number and viral gene expression in bovine monocytes. Monocytes (CD14^+^ cells) were isolated from PBMCs using MACS and transduced with either BAV304a or BAV888 using an MOI of 1. After 16 h, cells were collected and DNA and RNA were extracted. Total viral genome copy number recovered from each culture was quantified by qPCR (**A**). Expression of the *EYFP* gene and both early and late viral genes was quantified with qRT-PCR. Ct values for genes were normalized with β-actin (ΔCt) and presented as 1/ΔCt and compared for BAV304a- and BAV888-infected monocytes (**B**). Relative expression of EYFP and individual viral genes was compared for BAV304a (**C**) and BAV888 (**D**). Data presented are the mean + 1SD of values from three independent r experiments. * (*p* < 0.05); ** (*p* < 0.01); *** (*p* < 0.001); **** (*p* < 0.0001).

We then investigated whether the increased MFI for EYFP expression in BAV888-transduced monocytes was due to increased viral uptake. The DNA was purified from monocytes transduced with either BAV304a or BAV888 and viral DNA copy number was analyzed by quantitative real-time PCR, using BAdV-3-specific primers. A significantly (*p* < 0.05) higher viral genome copy number was detected in monocytes transduced with BAV888 versus BAV304a (Figure 7A). Consistent with the increased viral genome copy number in BAV888-transduced cells, there was also a significantly (*p* < 0.05) greater abundance of EYFP transcripts in BAV888-transduced monocytes when compared to BAV304a-transduced monocytes (Figure 7B).

### 3.9. Viral Gene Transcription in Transduced Monocytes (CD14+)

To determine if BAdV-3 gene transcription was also different in BAV304a- or BAV888-transduced monocytes, total RNA was isolated from MACS-purified CD14^+^ Bovine monocytes following infection with either BAV304a or BAV888. Viral gene expression was analyzed by qRT-PCR using viral gene-specific primers (Table 3). As seen in Figure 7B, there was a significant difference (*p* < 0.05) in the level of BAdV-3 19K and hexon-specific transcripts in monocytes transduced with BAV888 when compared to monocytes transduced with BAV304a. However, there was no significant difference in the level of BAdV-3 52K-specific transcripts in monocytes transduced with either BAV888 or BAV304a.

Interestingly, there were significant differences in the level of EYFP-specific transcripts compared to the level of BAdV-3-specific 19K (*p* < 0.01), 52K, and hexon (*p* < 0.001) transcripts in monocytes transduced with BAV304a (Figure 7C) and BAV888 (Figure 7D). Moreover, while, there were no significant difference in the level of BAdV-3 encoded 19K, 52K, and hexon gene-specific transcript in BAV304a-transduced monocytes (Figure 7C), there were significant difference in the level of BAdV-3-encoded 19K-specific transcripts compared to 52K (*p* < 0.05) and hexon (*p* < 0.01) specific transcripts in BAV888-transduced monocytes (Figure 7D).

## 4. Discussion

Recombinant human adenoviruses (HAdVs) can effectively deliver vaccine antigens in animals [3,31] but the large amount of recombinant HAdV-5 required to induce a protective immune response [31] is not compatible with the production of an economical vaccine for animals. Since non-human adenoviruses appear species specific, the development of species-specific adenovirus-based vectors may provide a better option for delivering vaccine antigens. This has led to the development and evaluation of BAdV-3 as vaccine-delivery vehicle for cattle [3]. However, developing more efficient and immunogenic BAdV-3 vectors requires knowledge of the normal interaction between wild-type BAdV-3 and bovine leukocytes, especially those cells involved in antigen presentation. One approach to increase the transduction of specific bovine leukocyte subpopulations is to modify the capsid proteins of the BAdV-3 vector [19,32]. Our current results reveal that BAdV-3 efficiently transduces bovine monocytes and neutrophils without detectable viral replication. Moreover, BAV888 (recombinant BAdV-3 expressing chimeric pIX fused to a RGD motif) increases virus uptake without significantly altering BAV304a (BAdV-3 expressing EYFP in E3 deleted region) tropism for monocytes.

The entry of HAdV-5 into the cells involves a primary interaction between fiber protein and the coxsackievirus-adenovirus receptor [CAR]) [33], and a secondary interaction between penton base “RGD” motif with α_ν_β_3_ and α_ν_β_5_ integrins on the cell surface [34]. However, little is known about the protein–protein interactions required for BAdV-3 entry into the cells. A primary interaction between BAdV-3 fiber with sialic acid has been reported [35] but the nature or existence of a secondary interaction has not been determined.

An earlier report suggested variable transduction of human lymphocytes by HAdV-11p and HAdV-35 [36]. BAV304a also appears to transduce a variety of bovine leukocytes, including PMN cells, with variable efficiency. While bovine monocytes and PMN cells were efficiently transduced by BAV304a, T cells, B cells, DCs, and ILCs were not efficiently transduced by BAV304a. It is possible, that the different levels of transduction observed among leukocyte subpopulations could be due to either the absence or presence of appropriate cell receptors. Similar transduction results were reported for human neutrophils, where the recombinant AdLuc (HAdV-5 expressing luciferase under the control of the CMV promoter) could transduce PMN cells, but could not express luciferase [37]. HAdV-5 vector uptake by neutrophils appeared to be independent of receptor-mediated binding. However, the expression of AdLuc within neutrophils may have been limited because of the presence of complement and antibodies in the human sera used for these assays [37]. In contrast, earlier studies reported successful transduction of bovine PMN cells by BAV304a [7,38]. Our study is consistent with these earlier results and while the transgene, under the control of CMV promoter was expressed, there was no detectable expression of BAV304a-specific genes. Since endogenous MHC class II expression has not been detected on bovine neutrophils, the expression of a transgene in bovine neutrophils may not contribute to the induction of adaptive immune responses [38]. Thus, eliminating BAdV-3 tropism for PMNs may be one strategy to improve the efficiency of antigen delivery by BAdV-3 vaccine vector. 

Previous studies reported that HAdV transduces human monocytes with the expression of E1A (E1) and hexon (L3) but no production of progeny virions was observed [39,40]. Like HAdV, BAV304a efficiently transduced bovine monocytes with the expression of 19K (E1), DBP (E2), and 52K (L1) but without the production of progeny virions. These results are consistent with the conclusion that adenoviruses establish abortive infections in leukocytes. Interestingly, an MOI of 10 resulted in significant reductions in both, the percent-transduced monocytes and the MFI of the EYFP protein suggesting that high levels of some BAV304a proteins may have an adverse effect on the monocyte viability and transgene expression.

Leukocytes, including antigen-presenting cells, have cell surfaces rich in integrins [28,29]. This creates the opportunity to use genetically modified adenovirus, containing a RGD motif inserted in the capsid proteins, to enhance the transduction of PBMCs [41]. Addition of the RGD motif to pIX in BAV888 did not significantly increase the percent CD14^+^ bovine monocytes transduced when compared to BAV304a. However, there was significant difference in the MFI for EYFP when comparing BAV888 to BAV304a-transduced monocytes. We speculate that the presence of the “RGD” motif may result in a secondary interaction between “RGD” and cellular integrins, thus modulating the BAV888 uptake. Several observations support this conclusion. First, viral genome copy number was significantly higher in BAV888 compared to BAV304a-transduced monocytes. Second, there was a significant difference in the abundance of EYFP and BAdV-3 19K (E1B^small^ region) transcripts in BAV888 compared to BAV304a-transduced monocytes. Third, the level of 19K-specific transcripts was significantly different than other BAdV-3-specific transcripts in monocytes transduced with BAV888 compared to monocytes transduced with BAV304a. Finally, unlike HAdV-5, the penton base protein of BAdV-3 does not contain the “RGD” motif [42] required for the secondary interaction between HAdV-5 and the host cell [34].

Monocytes are not considered professional antigen-presenting cells (APCs) but they can differentiate into macrophages or dendritic cells once they reach tissues. Once they differentiate into DCs, they can then effectively initiate immune responses [43,44]. Moreover, earlier studies suggest that immature monocytes themselves can act as antigen-presenting cells, processing antigens, retaining them, and presenting them to T cells in the lymph nodes after differentiation to monocyte-derived dendritic cells [45]. Therefore, targeting monocytes with BAdV3 may provide an efficient way to induce immune responses to proteins expressed as transgenes in BAdV-3-based vectors.

In summary, we demonstrated that among bovine leukocyte subpopulations, bovine monocytes and PMN cells are efficiently transduced by BAdV-3. Moreover, addition of an RGD motif in the BAdV-3 capsid (RGD motif in C-terminus of pIX and EYFP cassette in E3-deleted region) significantly enhanced the viral uptake by monocytes, without altering the viral tropism.

## Figures and Tables

**Table 1:** Monoclonal antibodies (MAbs) used to detect bovine leukocyte antigens.

Cell Surface Marker	Isotype	Catalog No	Supplier
CD14	IgG1	BOV2109	Washington State University
CD3	IgG1	BOV2009	Washington State University
CD21	IgG1	BOV2031	Washington State University
CD335	IgG1	BOV2147	Washington State University
CD209	IgG2a	BOV2133	Washington State University
MHCI	IgG2a	BOV2120	Washinton state University
MHCII	IgG2a	BOV2125	Washington State University
CD21	IgG1	MCA1424GA	AbD Serotec
CD11c	IgM	BAQ153A	VMRD
CD209	IgG2b	551186	BD Biosciences
CD335	IgG1	MCA2365	AbD Serotech
CD3	IgG1	MM1A	VMRD
CD14	IgG1	MM61A	VMRD

**Table 2:** List of secondary antibodies.

RReactivity	Fluorophore	Target IgG class	Catalog No	Supplier
Rat anti-mouse	APC	IgG1	17-4015-80	Thermo Fisher Scientific
Goat anti-mouse	APC	IgG2a	17-4010-82	Thermo Fisher Scientific
Rat anti-mouse	APC	IgM	550676	BD Biosciences
Goat anti-mouse	PE	IgG2b	P-21149	Invitrogen
Rat anti-mouse	APC	IgG1	560089	BD Pharmingen
Goat anti-mouse	APC	IgM	F0117	R & D systems

APC: allophycocyanin; PE: phycoerythrin.

**Table 3:** List of primers.

Target Gene	Direction	Sequence (5′ to 3′)
EYFP	Forward	AAGCTGACCCTGAAGTTCATCTC
	Reverse	CTTGTAGTTGCCGTCGTCCTTGAA
pVIII	Forward	CAGGTGCCAGTCAAGATTAC
BAdV-3	Reverse	ATGGCCGACTGAGTCATAAG
β-actin	Forward	GATCTGGCACCACACCTTCTAC
	Reverse	AGGCATACAGGGACAGCACA
Hexon	Forward	TGCTTCTTGCAAACACGACG
BAdV-3	Reverse	CCAATCTGAACCCCCGACAA
19 K	Forward	ATCGCACTGGAGTGTGGAAG
BAdV-3	Reverse	GGCACCACAAACACGTCAAA
52 K	Forward	ACCCTGGGTTTGATGCACTT
BAdV-3	Reverse	AGCTTCCCCAAAAATGCCCT
